# Multidisciplinary approach for patients with functional and non‐functional adrenal masses and review of the literature

**DOI:** 10.1002/hsr2.22

**Published:** 2017-12-20

**Authors:** Gamze Akkuş, Mehtap Evran, Murat Sert, Fesih Ok, Tamer Tetiker

**Affiliations:** ^1^ Internal Medicine and Endocrinology, Department of Internal Medicine, Division of Endocrinology Cukurova University Medical Faculty Adana Turkey; ^2^ Urology Department Cukurova Universty Medical Faculty Adana Turkey

**Keywords:** adrenal mass, hormone status, mass nature

## Abstract

**Background:**

Adrenal incidentalomas are adrenal masses that are discovered by imaging tests performed for other reasons.

**Aims:**

In this retrospective study, we analysed 229 Turkish patients with adrenal masses and who presented with or without complaints.

**Study design:**

Descriptive retrospective study and review of the literature

**Methods:**

This study conducted a retrospective review of 229 patients with adrenal incidentalomas that were referred to Cukurova University Hospital's endocrinological department between 2009 and 2014. We reviewed detailed patient histories, physical examination findings, and symptoms and signs related to hormonal hypersecretion or malignant neoplasm and recorded clinical indications for performing diagnostic radiological imaging. The statistical analysis of data was performed using SPSS‐19 software.

**Results:**

Of the 229 patients reviewed, 195 (85.2%) had non‐functional, benign adrenal adenomas, and 34 (14.8%) had functional lesions. Among those with functional lesions, 8 (3.5%) had lesions that secreted excess cortisol, 11 (4.8%) had lesions that secreted aldosterone, and 15 (6.6%) had lesions that secreted catecholamines. Eighty‐four patients included in the study (36.8%) underwent adrenalectomy; in 14 of these cases, the adrenalectomy was performed before surgical treatment criteria occurred. The most frequent pathologic diagnosis was adrenal cortical neoplasia (n = 38); 32 of these patients had adenomas (Weiss <4 criteria), and 6 had carcinomas (Weiss >4 criteria). Other patient diagnoses included benign pheochromocytoma (n = 13), pseudocyst (n = 12), metastasis (n = 10), haemorrhage (n = 3), necrosis (n = 1), hyperplasia (n = 2), and other (n = 5).

**Conclusions:**

Detailed endocrinological and radiological assessments of the mass nature and hormone status are necessary in cases of adrenal incidentaloma. Appropriate surgical treatment or periodic follow‐up must be determined based on the assessment results.

## 1 INTRODUCTION

The management of adrenal diseases has recently undergone major development as a result of rapid advances in laboratory and, in particular, imaging techniques. The available therapeutic options are also improving. If adrenal disease is suspected based on clinical findings, the diagnostic algorithm initially involves hormone tests to detect adrenal hyperfunction. These are followed by imaging studies addressing the morphological presentation of adrenal pathology, including evaluation of adjacent structures. Adrenal incidentalomas are adrenal masses that are discovered by imaging tests performed for other reasons.^1^ Today, in line with the increased use of computed tomography (CT) and magnetic resonance imaging (MRI), the prevalence of adrenal incidentaloma has increased, with a rate of 6% observed in autopsies or via imaging tests.^2^ The occurrence of adrenal incidentaloma increases with age: While the incidence rate is 1% for individuals under age 30, the rate climbs^3^ to 7% for people over age 70. Guidelines for the evaluation of adrenal incidentaloma have recently been published by the European Society of Endocrinology Clinical Practice Guideline in collaboration with the European Network for the Study of Adrenal Tumours.^4^ The first stage that must be assessed after diagnosing adrenal incidentaloma is an examination of the mass in terms of hormonal status and malignancy.^5^

Although the treatment of functional adrenal masses (Cushing syndrome, primary hyperaldosteronism [PHA], and pheochromocytoma) with clinical complaints is well‐known, novel clinical issues (subclinical Cushing syndrome [SCS]) require the most up‐to‐date treatment options. Although many similar studies^6^, ^7^ have been done in other endocrinology clinics, few studies of Turkish patients with adrenal masses have been conducted; accordingly, we aimed to retrospectively analyse 229 Turkish patients who had adrenal masses and who presented with or without complaints. Our emphasis was on the clinical characteristics of patients with adrenal masses and whether or not they were hormone‐active. In this article, we present a detailed report of the tumour pathology of the patients' adrenal masses, compare our clinical practice with the literature, and offer a different point of view by drawing attention to the fact that some patients who were followed up outside the endocrinology clinic were unnecessarily treated surgically.

## 2 MATERIALS AND METHODS

We performed a retrospective study of 229 patients with adrenal masses who were referred to the Cukurova University Hospital between 2009 and 2014. Cukurova University Hospital is a tertiary centre for patients in Turkey. The Local Ethics Committee of the University of Cukurova approved this study, and written informed consent was obtained from all subjects according to the Declaration of Helsinki. A retrospective study was conducted, and the patient data obtained included age (older than 15 y), sex, and anatomic characteristics of the adrenal mass (ie, size, location, endocrine function, and pathological findings from the patients' files). We separated age group of the our patients' as 15 to 44, 45 to 59, and +60 years.

Blood samples were collected at 8:00 am for the measurements of basal cortisol, adrenocorticotropic hormone (ACTH), dehydroepiandrosterone sulfate (DHEAS), and fasting blood glucose levels. Additionally, 24‐hour urinary cortisol excretion and midnight cortisol levels were measured, and 1‐mg dexamethasone suppression tests (DSTs) were recorded for all the patients. The diagnosis of SCS, in addition to cortisol levels greater than 1.8 mcg/dL after a 1‐mg DST,^8^ was based on the presence of at least one of the following: (1) urinary free cortisol levels of 300 mcg/day in 2 of the 3 consecutive collections per 24‐hour period, (2) ACTH values of <10 pg/mL, and (3) decreased DHEAS levels.

The same screening tests for pheochromocytoma were administered to all patients, including 24‐hour urinary tests for metanephrine and normetanephrine. If the 24‐hour urinary total metanephrine levels were higher than the references values, the mass was diagnosed as a pheochromocytoma.^9^ Particularly, in patients below the age of 40 who consulted for hypertension but were diagnosed with an adrenal mass, plasma‐renin activity (PRA) and plasma aldosterone values were recorded. Antihypertensive therapy (spironolactone and eplerenone) for these patients had been stopped 6 weeks earlier without aldosterone/renin ratio (ARR) examinations. In patients with a plasma ARR > 25, a PHA diagnosis was made via a saline infusion confirmatory test.^4^

Radiological findings related to the patients' adrenal lesions were interpreted by Cukurova University Hospital's radiology department. In terms of mass appearance per MRI, masses with regular contours, homogenous appearance, and showing fast washout during the in‐out phase after administration of the contrast agent were classified as “benign,” whereas masses with irregular contours, heterogeneous appearance, and not showing fast washout after administration of the contrast agent were classified as “malign.”^10^ In addition, we grouped MRI findings as 0 to 2, 2 to 4, 4 to 6, 6 to 8, 8 to 10, and >10 cm after the existence of the adrenal masses. Since the CT used in Cukurova University Hospital's radiology department does not address attenuation coefficients (Hounsfield unit), these values could not be recorded. The patients who had CT scans only were radiologically grouped as “benign” and “malign” according to mass size, contour irregularity, and the absence or presence of haemorrhage, necrosis, and calcification.^11^ Patients were examined using all imaging techniques performed at 6‐month intervals and grouped in terms of progression, regression, and stability of mass size. Patients with functional tumour, malignant characteristics as shown via MRI or CT, a mass diameter larger than 4 cm, or evidence of tumour growth during follow‐up imaging received surgical treatment.^4^

Pathological diagnoses were grouped as adrenal cortical neoplasia, pseudocyst, pheochromocytoma, haemorrhage, metastasis, necrosis, and other (sarcoma and myelipoma). Patients diagnosed with adrenal cortical neoplasia were reassessed according to Weiss criteria; thus, if 4 or more criteria were met, the mass was accepted as a carcinoma.^12^

DHEAS and ACTH values were studied with the enzymatic‐labelled chemiluminescent immunometric assay method (Immulite 2000; Diagnostic Products Corp., Los Angeles, CA, USA), whereas cortisol values were studied with chemiluminescence (Beckman DXI 800 autoanalyser). Plasma aldosterone concentrations were measured by Radioimmunoassay (RIA) and quantitative determination of PRA was done by the enzyme‐linked immunosorbent assay. Urine cortisol and metanephrine values were assessed via the high‐performance liquid chromatography method in which the container was acidified with 10 to 25 mL of 6 N HCl for the preservation of the metanephrines.

The statistical analysis of the data was performed using SPSS19.0 software. Descriptive statistics were performed for the demographic characteristics of the patient group. Categorical variables were evaluated via a chi‐square test, and continuous variables were evaluated with the Mann‐Whitney U test or analysis of variance test. Data were shown as mean ± standard deviation (mean ± SD); *P* < .05 was considered statistically significant.

## 3 RESULTS

### 3.1 Demographic characteristics

A total of 229 patients followed up for adrenal masses between 2009 and 2014 were included in our study. We found that female patients (n = 145, 66.3%) suffered more frequently from adrenal masses than male patients (n = 84, 36.7%). Our patients' older The mean patient age was 53.5 ± 12.2, and 29.3% patients were over the age of 60. When adenomas were evaluated in terms of incidence by age groups, it was found that patients aged 45 to 59 represented 48% of the patients.

### 3.2 Hormonal and metabolic parameters

An evaluation of all patients included in our study in terms of functionality of adrenal masses revealed a non‐functional mass rate of 85.2% (n = 195); 6 patients (2.6%) had CS, 11 patients (4.8%) had PHA, 15 patients (6.6%) had pheochromocytoma, and 2 patients (0.9%) had SCS.

The mean age of patients with a non‐functional mass (55 ± 11.7) was found to be significantly higher than the mean age of patients who had a functional mass (46 ± 12.50) (*P* < .001). In both groups, the number of female patients was higher than the number of male patients (*p* < .05).

Post high‐dose DST, the mean serum cortisol value of patients with CS was 24.42 ± 13.18 μ/dL, whereas the midnight cortisol value was 29.6 ± 6.62 μ/dL. The 24‐hour urine catecholamine results showed that urine metanephrine and normetanephrine values were 4 times higher than the normal values. The mean fasting plasma glucose of the patients was 111 ± 48.2 mg/dL, and the mean HbA1c value was 6.57% ± 1.57. Plasma lipid parameters (total cholesterol, HDL, LDL, and TG) and other biochemical parameters for all patients had not been performed because of the retrospective nature of the study.

### 3.3 Radiological characteristics

An examination of the imaging tests performed on the patients showed that CT and/or MRI frequencies were equal. It was observed that CT tests only or MRI tests only were performed on 12.2% of the patients (n = 28), whereas both CT and abdominal MRI tests were performed on 87.8% of the patients (n = 201). On the basis of the assumption that CT and MRI results are significantly more reliable, we did not take Ultrasound (USG) results into account in our study.

The demographic and clinical characteristics of the patients as well as the size, location, and functional features of the adrenal masses are presented in Table 1.

**Table 1:** Demographic and clinical characteristics of the patients as well as size, location, and functional features of adrenal masses (n = 229)

	Mean ± SD	n (%)
Age (years)		
15‐44	52 ± 22.7	52 (22.7%)
45‐59	110 ± 48	110 (48%)
60+	67 ± 29.3	67 (29%)
Mean	53.3 ± 12.2	
Gender		
Male		84 (63%)
Female		145 (36.7%)
Tumour size, cm	4.8 ± 2.26	229 (100%)
Location		
Unilateral		202 (88%)
Bilateral		27 (11%)
Accompanying disease status		
None		78 (34.1%)
DM (isolated)		16 (7%)
HT (isolated)		54 (23.6%)
CAD + HF		1 (4%)
Malignancy		22 (9.6%)
DM + HT		19 (19%)
DM + CAD		3 (1.3%)
HT + CAD		13 (5.7%)
Other		23 (10%)
Clinical Diagnoses		
Non‐functional		195 (85.2%)
Cushing syndrome		6 (2.6%)
PHA		11 (4.8%)
Pheochromocytoma		15 (6.6%)
SCS		2 (0.9%)
Carcinoma		6 (2.6%)
Histological Diagnosis		84 (36.6%)

Abbreviations: CAD, coronary artery disease; DM, diabetes mellitus; HF, heart failure; HT, hypertension; PHA, primary hyperaldosteronism; SCS, subclinical Cushing syndrome.

Unilateral adrenal masses were found in 88% of the patients (n = 202), whereas bilateral adrenal masses were found in 11% of the patients included in the study (n = 27).

In terms of mass size, 175 patients had masses <4 cm (76.4%), and 54 patients had masses >4 cm (23.2%). The patient mass sizes are shown in Table 2. In our study, patients were grouped based on having tumours with either a benign appearance or a malign appearance based on abdominal MRI findings. Accordingly, when we investigated whether there was a correlation between the MRI findings and the pathology results, we found that 5 of the 25 patients who were suspected of having malignant tumours based on MRI findings had not been surgically treated and that the remaining 20 patients (80%) had been surgically treated due to mass size and/or the presence of a functional mass. An examination of pathological findings revealed that MRI findings were parallel in 70% of the patients diagnosed with carcinoma metastasis and in 100% of the patients diagnosed with adrenocortical carcinoma (ACC) (Table 3).

**Table 2:** Correlations between mass size, clinical diagnosis, and functionality

Adrenal masses (n = 229)	Clinical diagnosis					
	Non‐functional	Cushing	PHA	Pheochromocytoma	SCS	Total
Adrenal Hyperplasia	20	1	0	0	0	**21**
**0‐2 cm**	68	1	3	3	1	**76**
**2‐4 cm**	68	3	3	4	0	**78**
**4‐6 cm**	20	1	2	7	1	**31**
**6‐10 cm**	15	0	1	1	0	**17**
**10+**	4	0	2	0	0	**6**
**Total**	**195**	**6**	**11**	**15**	**2**	**229**

Abbreviations: PHA, primary hyperaldosteronism; SCS, subclinical Cushing syndrome.

**Table 3:** Correlation between histopathological diagnosis, MR nature, and functionality

Histopathological diagnosis (n = 84)	MRI image		
	None, %	Suspected malignancy, %	No suspected malignancy, %
Pseudocyst (n = 12)	0	0	100
Haemorrhage (n = 3)	0	33.3	66.7
Necrosis (n = 1)	0	100	0
Hyperplasia (n = 2)	50	0	50
Pheochromocytoma (n = 13)	7.7	23.1	69.2
Adenoma (n = 32)	3.1	6.2	90.6
Carcinoma (n = 6)	0	100	0
Other (n = 15)	0	40	60
No histopathological diagnosis (n = 145)	15.2	3.4	81.4

### 3.4 Surgical treatment

Adrenalectomies had been performed on 36.8% of the patients (n = 84) in our study. Operational indications for the surgically treated patients were CS (n = 6), PHA (n = 11), (n = 15), SCS accompanied by deterioration in metabolic parameters (n = 1), tumour diameter > 4 cm, or having a suspicious appearance per radiology (n = 39). However, it was observed that 11 patients who had experienced adrenelectomies had undergone the procedures in the absence of any surgical indication, such as mass size or functionality.

### 3.5 Pathological diagnosis

The most frequently encountered pathological diagnosis was adrenal cortical neoplasia (n = 38, 45.2%). Thirty‐two of the patients with adrenal cortical neoplasia had adenomas (Weiss <4 criteria), and 6 had carcinomas (Weiss >4 criteria). Other diagnoses were, by order of frequency, benign pheochromocytoma (n = 13), pseudocyst (n = 12), haemorrhage (n = 3), necrosis (n = 1), hyperplasia (n = 2), and other, including myelolipoma, schwannoma, and ganglioneuroma (n = 15) (Table 3).

## 4 DISCUSSION

Many studies^6^, ^7^ on the characteristics of adrenal incidentalomas have been conducted in other countries. However, such studies on the Turkish population have been insufficient; we therefore wanted to describe our clinical experiences with adrenal masses, their hormonal status, surgery indications, and pathological diagnoses. In our study, we determined that 195 (85.2%) of the patients had non‐functional tumours and that the remaining patients (n = 34, 14.8%) had tumours that were functional. It is interesting to note that we found a higher prevalence of pheochromocytoma than previous reports of patients with adrenal incidentalomas at other clinics.^13^ Although we completed a thorough search for surgical indications of adrenal masses, we did not find any indications for 11 patients who had undergone adrenelectomies. Therefore, we also stress our belief that adrenal masses are important clinical issues that should be evaluated in a multidisciplinary manner.

Today, the term *adrenal incidentaloma* is a concept filled with question marks in terms of the distinctions between functional and non‐functional, benign and malign and surgical treatment versus follow‐up.^5^, ^14^ However, when an adrenal mass is encountered, the fundamental issues that must be addressed are whether or not the adrenal mass is functional and whether or not it is benign.^15^

In a series of 71 206 autopsy cases examined by Mantero et al,^16^ the prevalence of adrenal masses was reported to be 2.3% and, notably, was higher after the fifth decade.^17^ It was concluded that this result was a compensatory response to atherosclerosis that increased with advanced age and local ischaemic tissue dysfunction caused by atherosclerosis.^18^ In our study, 48.7% of the patients were over the age of 45. Previous studies have reported that adrenal incidentaloma was higher in the right adrenal gland and in the female sex and was bilateral in only 10% to 15% of the cases.^15^ The finding related to gender is thought to be associated with the fact that female patients visit doctors much more frequently for any reason and undergo many more scans and imaging in particular. In our study, the number of female patients was higher (62.9%) than the males (37.1%), and 11.8% of the masses were bilateral.

The most important phase in relation to adrenal incidentalomas is the one in which tests performed to determine whether or not a tumour is hormone‐active are evaluated after the diagnosis. Previous studies reported that 70% of these adenomas were non‐functional, 8% to 25% consisted of cortisol‐secreting adenomas with (CS), 1% consisted of aldosterone‐secreting adenomas (Conn syndrome), and 5% had pheochromocytoma.^13^, ^19^ Moreover, there is evidence suggesting that tumour size is correlated with its functionality. It was found that the bigger the tumour size, the more hormones—notably cortisol—are secreted.^20^ Similarly, most of the patients in our study had non‐functional adrenal masses, 19.6% of which were >4 cm; additionally, 44.2% the functional masses were >4 cm.

Although it is known that pheochromocytomas had a prevalence of 4% to 7% among adrenal incidentalomas, that prevalence was observed to climb to 20% in some studies.^21^, ^22^ Although there has been no clear consensus to date, the fact that urine fractional metanephrine values have a higher sensitivity and specificity than plasma metanephrine values have led this method to be accepted as the gold standard for diagnosis.^23^, ^24^ In our study, all the patients with pheochromocytoma had urine metanephrine values that were as much as 4 times the normal levels. Primary hyperaldosteronism is a disease that is notable for its prevalence of 10% among hypertensive patients and its prevalence of 1.5% to 7% among patients with adrenal incidentalomas.^25^ Unlike the long‐known comorbidity of hypertension and hypokalaemia, some of the patients who are diagnosed today are seen to be normotensive and normokalemic.^26^ The most important test for diagnosis at the first stage is evaluation of plasma aldosterone (ng/dL) and PRA rates (ng/mL/h).^27^ Of the patients with PHA in our study (36.3%), 4.8% (n = 4) were found to be normotensive during physical examination, and only 2 patients (18.2%) were hypokalaemic. ARR was found to be >25 in our patients.

Subclinical Cushing syndrome, which particularly causes increased insulin resistance, type 2 diabetes mellitus, obesity, and osteoporosis, is the most frequently encountered hormonal dysfunction in adrenal incidentalomas.^8^ This is a term used to describe that cortisol production is insufficient to cause clinically recognizable syndrome and to suppress the release of corticotropin releasing hormone and ACTH. In previous studies, the SCS prevalence was reported to be 5% to 20% in patients with adrenal incidentalomas.^28^ Because in our study, some of the patients with an adrenal mass were followed up by other clinics and serum cortisol, ACTH and 24‐hour urine cortisol values of these patients could not be obtained, we think that the number of patients with SCS might possibly be higher than the rate identified (0.9%). It is obvious that further prospective studies on this subject are needed.

The aetiology of adrenal masses, including lipomas, myelolipomas, neurofibromas, schwannomas, haemangiomas, leiomyosarcomas, infections, granulomas, infiltrations, cysts, pseudocysts, and metastases constitute the “others” group and is pathologically diverse.^15^, ^29^ Weiss criteria are used to facilitate benign and malign classifications as well as an understanding of masses rooted from the adrenal cortex.^8^ According to these criteria, the presence of more than 4 of the criteria, including high nuclear grade, mitosis >2 (×50), atypical mitoses, 25% or less transparent cell, diffuse structure, necrosis, venous invasion, sinusoidal invasion, and capsular invasion, are positive, suggests malignancy.^30^ In a study conducted by Barzon et al in 2000, adrenal adenomas, nodular hyperplasia, carcinoma, pseudocysts, metastases, and pheochromocytomas were found at a rate of 36% to 84%, 7% to 17%, 1.2% to 11%, 4% to 22%, 0% to 22%, and 1.5% to 23%, respectively, in the pathological diagnoses of adrenal incidentalomas.^20^ The pathology results of adrenal masses in our study are similar to the findings of these previous studies.

Adrenocortical cancer (ACC) has an incidence of 1 to 2 per million and is responsible for 0.2% of all deaths associated with cancer. In ACC, tumour size is generally 4 to 6 cm and above.^31^, ^32^ In our study, the ACC rate was 2.62%; 5 of these patients were found to have non‐functional tumours, and one had aldosterone‐secreting carcinoma. The average size of 6 carcinomas was found to be 8.1 ± 2.1 cm.

As previously mentioned, the second question that must be answered in adrenal incidentalomas is whether or not the mass is malignant. From imaging methods, particularly CT and MRI, we know that the differences in appearance caused by the high lipid content of benign lesions give us information at the first stage.^33^ Previous studies^11^, ^34^ have shown that an adrenal adenoma is seen in CT as a round mass, sized <4 cm, with regular contours, homogenous appearance, and a high lipid content; carcinoma appears as a mass sized >4 to 6 cm, with irregular contours, heterogeneous appearance, occasionally containing calcification and in some patients, a mass with invasion into surrounding tissues.^11^, ^35^ Magnetic resonance imaging, another imaging method used to examine adrenal incidentalomas, is known not to be much different than CT in terms of diagnosis and offers a sensitivity of 78% and a specificity of 87%.^36^ In general, normal adrenal tissues and adenomas appear with low‐signal intensity in T1‐ and T2‐weighted sequences, whereas malignant lesions appear hypointense in T1‐weighted sequences and hyperintense in T2‐weighted sequences.^37^ Identical to a study by Musella,^38^ we found postsurgery pathological diagnoses of masses that were suspected to be malignant based on CT or MRI findings and were found to be ACC (100%) or carcinoma metastasis (70%), in line with the imaging results.

According to the European Society of Endocrinology Clinical Practice Guideline in collaboration with the European Network for the Study of Adrenal Tumours,^4^ the treatment algorithm recommended for adrenal incidentalomas is surgical treatment if the mass is hormone‐active (ie, functional), is sized >4 to 6 cm, and there are parameters that suggest malignancy in imaging (contour irregularity, heterogeneity, haemorrhage, central necrosis, and calcification). In our study, 6 patients were surgically treated due to CS, 11 patients due to PHA, 15 patients due to pheochromocytoma, 1 patient due to SCS with deterioration in metabolic parameters, and 38 patients due to progression in non‐functional masses. No surgical indication was found in 11 patients who had undergone adrenelectomies, although they had non‐functional masses sized <4 cm and had no malignancy findings in imaging studies. Nonetheless, 2 of these patients developed postsurgical complications, such as pancreatic fistula and wound site infections.

In summary, the approach to adrenal masses in patients with or without complaint should be investigated using a multidisciplinary approach. Particularly, after detailed endocrinological and radiological assessments of the mass nature and hormone status, treatment or follow‐up should be clarified. Unnecessary surgical treatments can thus be avoided, and important benefits achieved in terms of the patient, the doctor, and the national economy.

## 5 LIMITATIONS

This study has some limitations, such as a small sample size, lack of the Hounsfield unit (HU) in CT imaging and missing patients' data due to retrospective design.

## CONFLICT OF INTEREST

None declared.
